# RNA-seq analysis of short fiber mutants Ligon-lintless-1 (*Li*_*1*_) and – 2 (*Li*_*2*_) revealed important role of aquaporins in cotton (*Gossypium hirsutum* L.) fiber elongation

**DOI:** 10.1186/s12870-015-0454-0

**Published:** 2015-02-27

**Authors:** Marina Naoumkina, Gregory N Thyssen, David D Fang

**Affiliations:** Cotton Fiber Bioscience Research Unit, USDA-ARS-SRRC, 1100 Robert E. Lee Blvd, New Orleans, LA 70124 USA

## Abstract

**Background:**

Cotton fiber length is a key determinant of fiber quality for the textile industry. Understanding the molecular basis of fiber elongation would provide a means for improvement of fiber length. Ligon lintless-1 (*Li*_*1*_) and Ligon lintless-2 (*Li*_*2*_) are monogenic and dominant mutations, that result in an extreme reduction in the length of lint fiber to approximately 6 mm on mature seeds. In a near-isogenic state with wild type (WT) cotton these two short fiber mutants provide an excellent model system to study mechanisms of fiber elongation.

**Results:**

We used next generation sequencing (RNA-seq) to identify common fiber elongation related genes in developing fibers of *Li*_*1*_ and *Li*_*2*_ mutants growing in the field and a greenhouse. We found a large number of differentially expressed genes common to both mutants, including 531 up-regulated genes and 652 down-regulated genes. Major intrinsic proteins or aquaporins were one of the most significantly over-represented gene families among common down-regulated genes in *Li*_*1*_ and *Li*_*2*_ fibers. The members of three subfamilies of aquaporins, including plasma membrane intrinsic proteins, tonoplast intrinsic proteins and NOD26-like intrinsic proteins were down-regulated in short fiber mutants. The osmotic concentration and the concentrations of soluble sugars were lower in fiber cells of both short fiber mutants than in WT, whereas the concentrations of K^+^ and malic acid were significantly higher in mutants during rapid cell elongation.

**Conclusions:**

We found that the aquaporins were the most down-regulated gene family in both short fiber mutants. The osmolality and concentrations of soluble sugars were less in saps of *Li*_*1*_ – *Li*_*2*_, whereas the concentrations of malic acid, K^+^ and other detected ions were significantly higher in saps of mutants than in WT. These results suggest that higher accumulation of ions in fiber cells, reduced osmotic pressure and low expression of aquaporins, may contribute to the cessation of fiber elongation in *Li*_*1*_ and *Li*_*2*_ short-fiber mutants. The research presented here provides new insights into osmoregulation of short fiber mutants and the role of aquaporins in cotton fiber elongation.

**Electronic supplementary material:**

The online version of this article (doi:10.1186/s12870-015-0454-0) contains supplementary material, which is available to authorized users.

## Background

Cotton is the major source of natural fibers used in the textile industry. Apart from its economic importance, the cotton fiber provides a unique single-celled model system to study cell elongation and cell wall biogenesis in the absence of cell division [1]. Cotton fiber development consists of four distinct but overlapping stages, including fiber initiation, elongation, secondary cell wall biosynthesis, and maturation [1]. Each cotton fiber is a single cell that initiates from the epidermis of the outer integument of the ovules at or just prior to anthesis [2]. Fiber elongation starts on the day of anthesis and continues for about 3 weeks before the cells switch to intensive secondary cell wall cellulose synthesis. Lint fibers of the economically important *Gossypium hirsutum* generally grow about 30–40 mm in length. During peak elongation fiber cells can increase in length at rates of 2 mm per day or more depending on environment and genotype [1-3]. The fiber cells elongate up to 3000 fold during 3 weeks which makes them the fastest growing and longest single cell known in higher plants [4]. Understanding the molecular basis of fiber elongation would provide a means for cotton breeders and researchers to improve the fiber length while maintaining yield and other cotton characteristics.

Genetic mutants are useful tools for studying the molecular mechanisms of fiber development. Our laboratory uses two short fiber mutants, Ligon lintless-1(*Li*_*1*_) and Ligon lintless-2 (*Li*_*2*_) as a model system to study fiber elongation [5-10]. Both *Li*_*1*_ and *Li*_*2*_ are monogenic and dominant mutations, resulting in an extreme reduction in the length of lint fiber to approximately 6 mm on mature seeds [11,12]. Both mutations are located in the D_T_ subgenome of *G. hirsutum*: the *Li*_*1*_ gene is on chromosome 22 [8,13,14], whereas the *Li*_*2*_ gene is on chromosome 18 [5,10,14,15]. Cytological studies of cotton ovules did not reveal much difference between mutants and their near-isogenic WT lines during initiation and early elongation up to 3 DPA [5,13]. In a fiber developmental study Kohel and co-authors observed that the elongation pattern is similar and restricted in both, *Li*_*1*_ and *Li*_*2*_ fibers [16]. However, unlike the normal morphological growth of the *Li*_*2*_ plants, the *Li*_*1*_ mutant exhibits pleiotropy in the form of severely stunted and deformed plants in both the homozygous dominant and heterozygous state [8,11,12]. The near-isogenic lines (NILs) of *Li*_*1*_ and *Li*_*2*_ with the elite Upland cotton variety DP5690 previously used in our research [5,8] provide an excellent model system to study mechanism of fiber elongation.

In our previous report we used a microarray approach to identify common genes related to fiber elongation, those with altered expression as a result of the *Li*_*1*_ and *Li*_*2*_ mutations, growing in the field and a greenhouse [7]. We found a relatively small number; 88 genes were differentially regulated in both short fiber mutants, which may be due to limitations of microarray technology. RNA-seq offers a larger dynamic range of quantification, reduced technical variability, and higher accuracy for distinguishing and quantifying expression levels of homeologous copies than microarray [17]. RNA-seq can provide a more comprehensive and accurate transcriptome analysis of cotton fiber development by using the reference genome sequence of *Gossypium raimondii* Ulbr. [18].

In this study we used a RNA-seq approach for the same goal: to determine fiber elongation related genes affected in both mutants growing in the field and a greenhouse. We found a larger number of differentially regulated genes common to both mutants, and from those the major intrinsic proteins were significantly over-represented among the down regulated genes. We measured the osmolality and concentrations of major osmotic solutes in sap of fiber cells. Although the osmolality and the concentrations of soluble sugars were less in saps of both short fiber mutants than in WT the concentrations of K^+^ and malic acid were significantly higher in saps of mutants than in WT during rapid elongation time. The higher concentrations of malic acid and ions suggest limited uptake of water into fiber cells of mutants that can be result of down regulation of major intrinsic proteins.

## Results

### Sources of variability in RNA-seq data

We examined genome-wide gene expression in elongating cotton fiber cells at 8 DPA in *Li*_*1*_, *Li*_*2*_ mutants and WT under different growing conditions, in the field and greenhouse. The time point 8 DPA was selected because our earlier research revealed significant transcript and metabolite changes between the *Li*_*2*_ and WT NILs during this time of fiber development [5,6]. Approximately 1.06 billion 100 bp reads from 13 libraries, including 9 libraries from field grown plants (this work) and 4 libraries from greenhouse grown plants (previously reported [9]), were trimmed with Sickle [19] and mapped to transcripts from the *G. raimondii* genome reference sequence [18,20]. The results of mapping reads are provided in Additional file 1.

**Figure 1:**
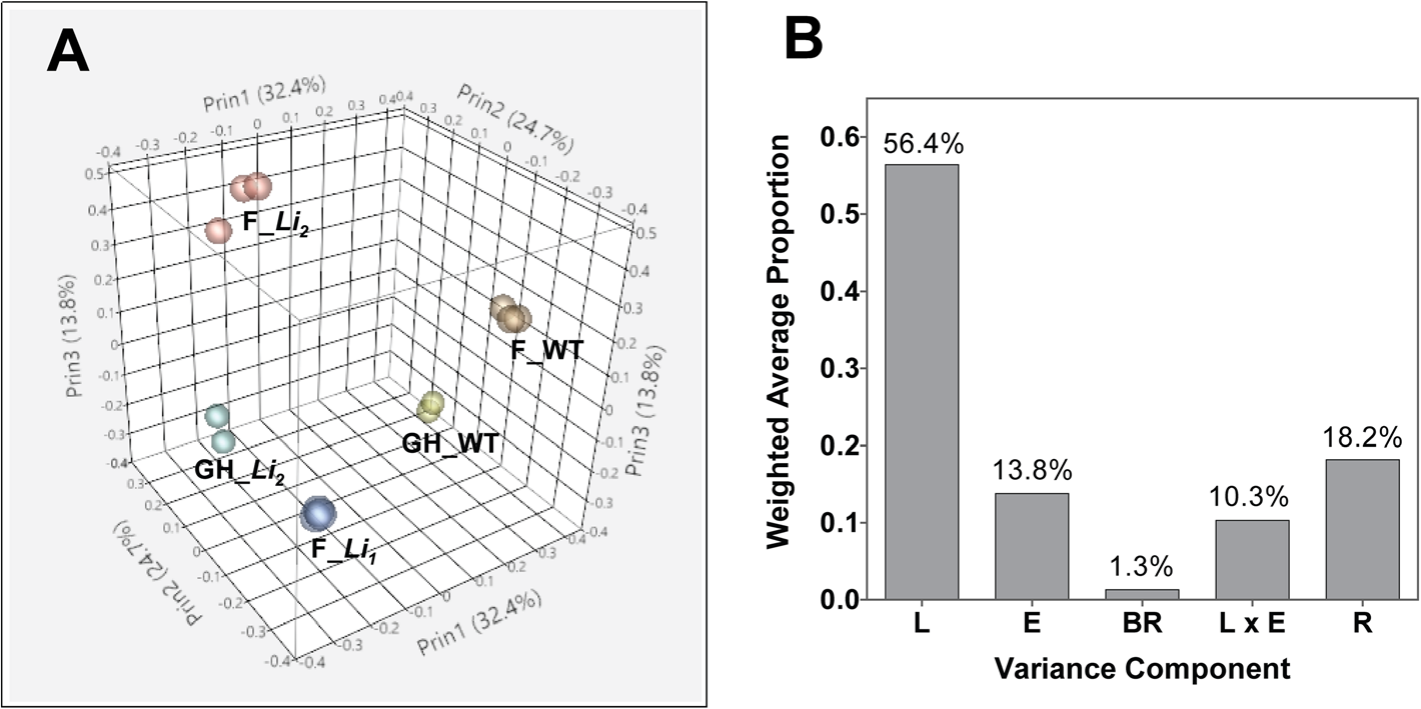
**Sources of variability in RNA-seq data. (A)** Principal component analysis of RNA-seq samples from developing fibers (at 8 DPA) of *Li*_*1*_, *Li*_*2*_ and WT NILs. F: field grown plants; GH: greenhouse grown plants. **(B)** Proportion of the transcriptional variance explained by each variance component. L: near-isogenic lines, *Li*_*1*_, *Li*_*2*_ and WT; E: environmental factors, greenhouse and field; BR: biological replicates; and R: residual.

Principal component analysis (PCA) was applied to explore relationships in gene expression among the samples. According to PCA, the samples from the near-isogenic lines and from the same lines growing in the field and a greenhouse are separated, indicating effects of the mutations and growth conditions on gene expression (Figure 1A). To further investigate the proportion of variation in gene expression explained by each factor, a principal variance components analysis (PVCA) was run on the same data set. This approach first reduces data dimensionality with PCA, and then fits a mixed linear model to each principal component with variance components analysis (VCA). The largest source of variability in fiber transcriptome was the variance component L (the near-isogenic lines; weighted average proportion of 56.4%), whereas the variance component E (environmental factor) explained 13.8% of the total transcriptional variance (Figure 1B).

### Differential gene expression analysis

**Figure 2:**
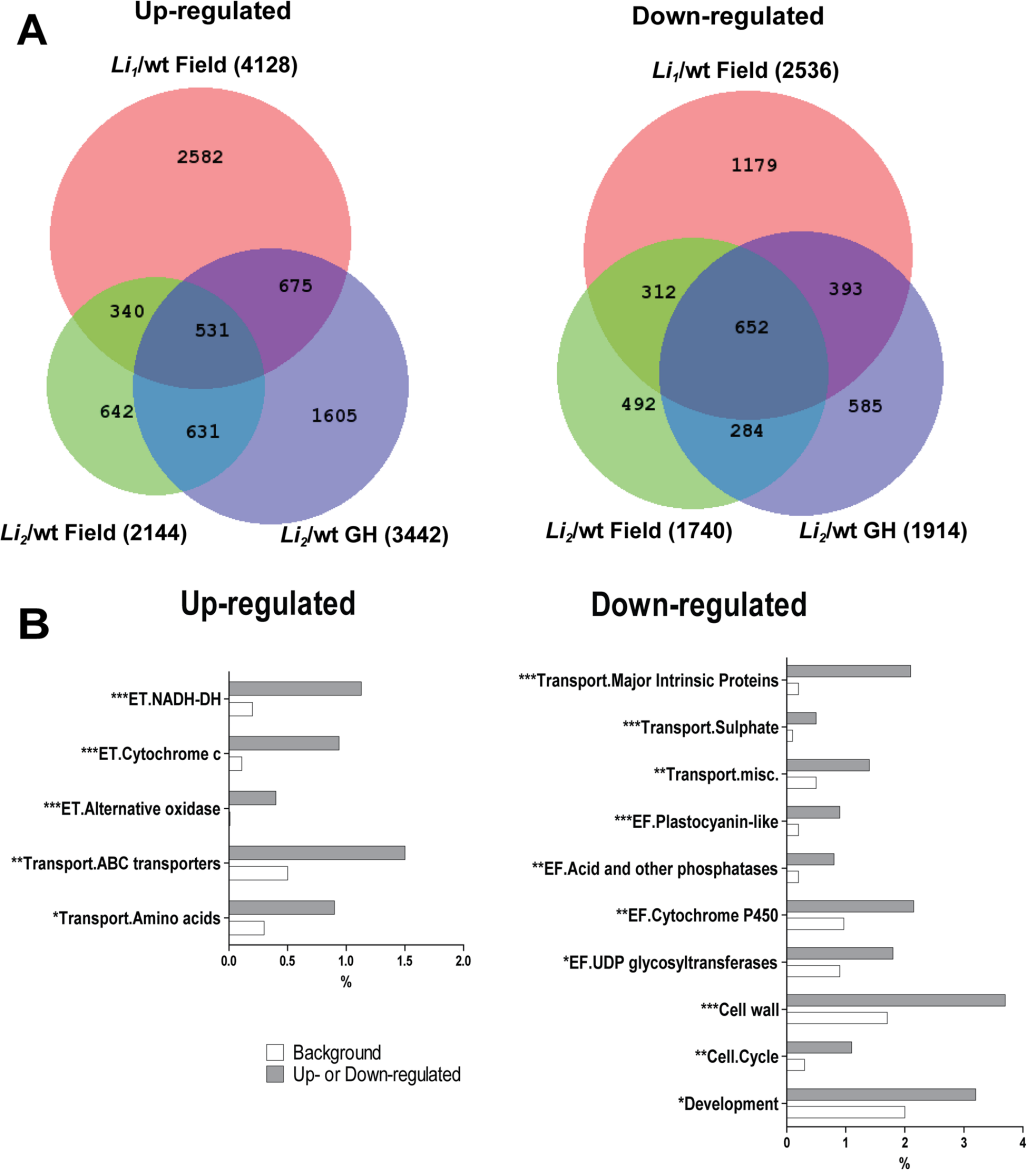
**Overview of differentially expressed genes in developing fibers of mutants comparing with WT under different growth conditions. (A)** Venn diagrams of significantly up-regulated genes (left) and down-regulated genes (right) in *Li*_*1*_/wt and *Li*_*2*_/wt grown in field and greenhouse (GH). Total number of significantly regulated genes in each comparison is indicated in parentheses. **(B)** Gene set enrichment analysis of common regulated genes among short fiber mutants grown in field and greenhouse. As indicated in section **(A)** of this figure there are 531 up-regulated and 652 down-regulated common genes. MapMan BIN structure was used for functional categorization of common regulated genes. Shown are only the significantly overrepresented subcategories; the number of asterisks indicate the level of significance (i.e. *p < 0.05, **p < 0.001). Relative gene frequencies in functional categories are presented in percents from amount of up-regulated or down-regulated genes; background represents pseudo-*G. hirsutum* genome generated by doubling the reference *G. raimondii* genome. Abbreviations: ET, electron transport; and EF, miscellaneous enzyme families.

An ANOVA model for gene expression was specified in which the measured level of gene expression in *Li*_*1*_ and *Li*_*2*_ under different growth conditions was compared with gene expression in corresponding WT. The ANOVA analysis of transcript data is provided in Additional file 2. We found that 4,128 genes were significantly (FDR q-value < 0.05) up-regulated in field grown *Li*_*1*_ fibers, whereas only 2,144 genes were up-regulated in field grown *Li*_*2*_ fibers and 3,442 genes were up-regulated in greenhouse grown *Li*_*2*_ fibers (Figure 2A). The largest amount of down-regulated genes 2,536 was detected in field grown *Li*_*1*_ fibers, whereas 1,740 and 1,914 genes were down-regulated in field and greenhouse grown *Li*_*2*_ fibers, consequently. Only small portions of these genes were common among up-regulated (531) and down-regulated (652) in all tested conditions by ANOVA model (Figure 2A). In the following gene set enrichment analysis we focused only on these common genes since our objective was to identify fiber elongation related genes common between short fiber mutants growing in the field and a greenhouse.

MapMan ontology was used for gene set enrichment analysis [21]. Two main categories (electron transport and transport) were overrepresented among up-regulated genes and five main categories (transport, enzyme families, cell wall, cell and development) were overrepresented among down-regulated genes in *Li*_*1*_- *Li*_*2*_ developing fibers. Figure 2B shows only sub-categories from the above mentioned main categories which are significantly (Chi-square, p < 0.05) overrepresented in the *Li*_*1*_ – *Li*_*2*_ fiber transcriptomes. Particularly, NADH dehydrogenase, cytochrome c and alternative oxidase were significantly (p < 0.0001) overrepresented sub-categories in electron transport, whereas ABC transporters and transport of amino acids were overrepresented sub-categories *Li*_*1*_ – *Li*_*2*_ up-regulated genes. The most significantly (p < 0.0001) overrepresented sub-categories in *Li*_*1*_ – *Li*_*2*_ down-regulated genes were: major intrinsic proteins and transport of sulphate in transport category; and the plastocyanin –like enzyme family.

Genes categorized into transport functional category were overrepresented among up-regulated and down-regulated pools of genes; however, proportions of gene family members of transporters were different among up-regulated or down-regulated genes. Significantly up-regulated and down-regulated transporters in *Li*_*1*_ – *Li*_*2*_ mutants growing in the field and a greenhouse are shown in Tables 1 and 2. Major intrinsic proteins, sulphate and phosphate transporters were present only among pool of down-regulated genes, whereas proportions of amino acids and ABC transporters were significantly higher among pool of up-regulated genes. The sugars transporters were not significantly more abundant among up-regulated than down-regulated genes.

**Table 1:** **Significantly up-regulated transporters in** ***Li***_***1***_ **and** ***Li***_***2***_ **mutants regardless of growth conditions**

**Gene-subgenome/subcategory**	***Li***_***1***_ **/wt F**	***Li***_***2***_ **/wt F**	***Li***_***2***_ **/wt GH**	**Description**
**Sugars**				
Gorai.007G292300_A	2.9	1.5	1.7	sugar:hydrogen symporter
Gorai.012G130400_A	2.5	2.3	1.5	mannitol transporter
Gorai.011G046300_D	1.7	1.6	1.9	inositol transporter 2
Gorai.005G139700_D	2.6	1.6	1.7	sucrose transporter 2
**Amino acids**				
Gorai.009G126900_D	2.0	1.1	1.0	Inorganic H pyrophosphatase family
Gorai.013G148800_A	3.5	1.8	2.3	amino acid permease 7
Gorai.002G233100_D	1.0	1.3	2.1	aromatic and neutral transporter 1
Gorai.005G253300_D	1.3	1.4	1.2	amino acid permease
Gorai.013G148600_A	2.2	1.8	1.6	amino acid permease 7
**Metabolite transporters at the envelope membrane**				
Gorai.007G313700_D	1.3	1.2	1.3	phosphate translocator 1
**NDP-sugars at the ER**				
Gorai.008G116300_A	4.2	2.1	1.4	UDP-galactose transporter 2
**Metal**				
Gorai.007G173100_A	2.7	2.2	2.4	zinc transporter 5 precursor
**Peptides and oligopeptides**				
Gorai.004G290200_D	4.3	3.4	4.1	Major facilitator superfamily protein
Gorai.008G190200_D	2.4	1.3	1.7	Major facilitator superfamily protein
**Unspecified cations**				
Gorai.010G187200_D	3.0	1.3	1.8	tonoplast dicarboxylate transporter
Gorai.012G145500_A	1.4	1.1	1.0	Magnesium transporter CorA-like family
**Potassium**				
Gorai.009G055600_D	2.5	1.4	1.5	K+ channel tetramerisation domain
**ABC transporters**				
Gorai.007G310800_A	2.2	2.6	1.3	multidrug resistance-associated protein 3
Gorai.007G310800_D	2.8	2.9	2.0	multidrug resistance-associated protein 3
Gorai.007G310700_D	2.4	2.9	2.3	multidrug resistance-associated protein 3
Gorai.007G310600_A	2.3	2.6	1.4	multidrug resistance-associated protein 3
Gorai.007G310600_D	3.0	2.9	2.7	multidrug resistance-associated protein 3
Gorai.001G003100_D	1.7	2.8	1.9	pleiotropic drug resistance 10
Gorai.013G154800_D	1.3	2.2	1.6	multidrug resistance-associated protein 3
Gorai.007G070500_D	2.0	1.7	1.2	multidrug resistance protein
**Calcium**				
Gorai.007G021200_D	1.5	1.4	1.4	CAX interacting protein 1
Gorai.013G148900_D	1.8	2.3	2.0	cation exchanger 2
**Miscellaneous**				
Gorai.009G306300_D	1.6	1.7	1.2	Auxin efflux carrier family protein
Gorai.013G014100_D	2.8	3.1	2.8	Auxin efflux carrier family protein
Gorai.005G179100_A	2.3	1.2	1.7	MATE efflux family protein
Gorai.013G170200_A	2.6	1.8	1.3	MATE efflux family protein
Gorai.009G171800_A	1.8	1.0	1.1	secretory carrier 3
Gorai.009G208500_D	2.0	1.4	1.2	Xanthine/uracil permease family protein
Gorai.009G208500_A	2.3	1.3	1.3	Xanthine/uracil permease family protein

Numbers represent the log base 2 ratio of mutants to wild-type expression; F, field grown plants; and GH, greenhouse grown plants.

**Table 2:** **Significantly down-regulated transporters in** ***Li***_***1***_ **and** ***Li***_***2***_ **mutants regardless of growth conditions**

**Gene-subgenome/subcategory**	***Li***_***1***_ **/wt F**	***Li***_***2***_ **/wt F**	***Li***_***2***_ **/wt GH**	**Description**
**Sugars**				
Gorai.009G135300_D	−1.2	−1.3	−1.6	carbohydrate transmembrane transporter
Gorai.010G030700_D	−1.5	−1.0	−1.2	sucrose transporter 4
**Amino acids**				
Gorai.006G146500_A	−2.1	−1.4	−3.9	aromatic and neutral transporter 1
Gorai.006G146500_D	−2.0	−1.6	−2.4	aromatic and neutral transporter 1
Gorai.009G453900_A	−1.3	−1.6	−1.7	amino acid permease 7
Gorai.009G321600_A	−1.0	−1.0	−1.2	proline transporter 2
**Sulphate**				
Gorai.002G059100_A	−1.9	−1.1	−2.5	sulfate transporter 3;4
Gorai.002G059100_D	−2.0	−1.2	−2.0	sulfate transporter 3;4
Gorai.009G240100_A	−2.8	−1.2	−1.8	STAS domain/Sulfate transporter family
**Phosphate**				
Gorai.008G179500_A	−1.5	−1.2	−3.0	EXS (ERD1/XPR1/SYG1) family protein
Gorai.010G140300_A	−1.3	−1.3	−1.4	phosphate transporter 1;7
**Metabolite transporters at the envelope membrane**				
Gorai.004G292400_A	−1.2	−1.3	−1.2	Nucleotide-sugar transporter family protein
Gorai.008G241700_A	−1.9	−1.1	−1.5	Nucleotide-sugar transporter family protein
Gorai.003G043000_D	−1.7	−1.7	−1.3	uncoupling protein 5
**Metal**				
Gorai.011G049700_D	−3.4	−1.6	−1.8	zinc transporter 10 precursor
Gorai.003G073800_D	−2.0	−1.2	−1.1	Cation efflux family protein
**Peptides and oligopeptides**				
Gorai.007G049100_D	−1.3	−1.9	−1.4	oligopeptide transporter 7
Gorai.007G049100_A	−1.3	−2.1	−1.4	oligopeptide transporter 7
Gorai.009G271300_A	−2.4	−1.5	−1.9	peptide transporter 1
**Unspecified cations**				
Gorai.006G257200_D	−3.9	−3.4	−3.7	sodium hydrogen exchanger 2
Gorai.002G024800_D	−2.4	−2.7	−1.6	magnesium transporter 9
**Potassium**				
Gorai.010G066400_A	−1.2	−1.2	−1.7	K+ transporter 1
**ABC transporters**				
Gorai.009G304900_A	−1.2	−1.2	−1.3	pleiotropic drug resistance 6
Gorai.002G162300_A	−2.6	−1.9	−3.2	non-intrinsic ABC protein 12
Gorai.001G057400_D	−1.6	−1.6	−2.1	pleiotropic drug resistance 12
Gorai.003G062100_D	−1.5	−1.1	−3.6	ABC-type transporter family protein
**Major intrinsic proteins**				
Gorai.004G001400_D	−3.7	−2.9	−5.1	plasma membrane intrinsic protein 2;4
Gorai.002G002500_A	−2.3	−1.9	−2.5	plasma membrane intrinsic protein 3
Gorai.002G002500_D	−2.0	−1.6	−2.1	plasma membrane intrinsic protein 3
Gorai.002G248400_D	−3.1	−2.6	−2.4	plasma membrane intrinsic protein 2
Gorai.002G248400_A	−1.9	−1.7	−2.1	plasma membrane intrinsic protein 2
Gorai.004G212800_A	−1.3	−1.2	−1.9	plasma membrane intrinsic protein 1;4
Gorai.004G212800_D	−1.4	−1.1	−1.8	plasma membrane intrinsic protein 1;4
Gorai.011G098100_D	−3.1	−1.9	−3.4	plasma membrane intrinsic protein 3
Gorai.011G098100_A	−2.9	−1.6	−1.7	plasma membrane intrinsic protein 3
Gorai.013G265400_D	−2.4	−1.3	−2.1	tonoplast intrinsic protein 1;3
Gorai.013G265400_A	−2.1	−1.4	−1.9	tonoplast intrinsic protein 1;3
Gorai.002G245900_A	−2.7	−2.4	−3.4	gamma tonoplast intrinsic protein
Gorai.003G136600_A	−2.9	−2.7	−3.2	tonoplast intrinsic protein 1;3
Gorai.007G078800_A	−2.3	−1.2	−2.5	NOD26-like intrinsic protein 4;2
**Calcium**				
Gorai.013G196000_D	−1.4	−1.3	−1.4	Sodium/calcium exchanger family protein
**Miscelleneous**				
Gorai.003G000900_D	−3.2	−2.9	−4.0	Auxin efflux carrier family protein
Gorai.003G000900_A	−4.8	−3.9	−3.9	Auxin efflux carrier family protein
Gorai.008G124800_D	−1.1	−1.0	−1.4	cyclic nucleotide gated channel 1
Gorai.007G212500_A	−1.3	−2.3	−2.2	MATE efflux family protein
Gorai.007G212500_D	−1.7	−2.8	−1.8	MATE efflux family protein
Gorai.002G230300_D	−1.4	−1.8	−1.9	MATE efflux family protein
Gorai.001G084300_D	−1.9	−2.2	−2.6	MATE efflux family protein
Gorai.003G066500_A	−4.0	−3.1	−2.7	phosphoglyceride transfer family protein
Gorai.002G017400_A	−2.1	−1.6	−3.0	Secretory carrier membrane protein
Gorai.002G017400_D	−1.2	−1.3	−1.9	Secretory carrier membrane protein

Numbers represent the log base 2 ratio of mutants to wild-type expression; F, field grown plants; and GH, greenhouse grown plants.

### Major intrinsic proteins

**Figure 3:**
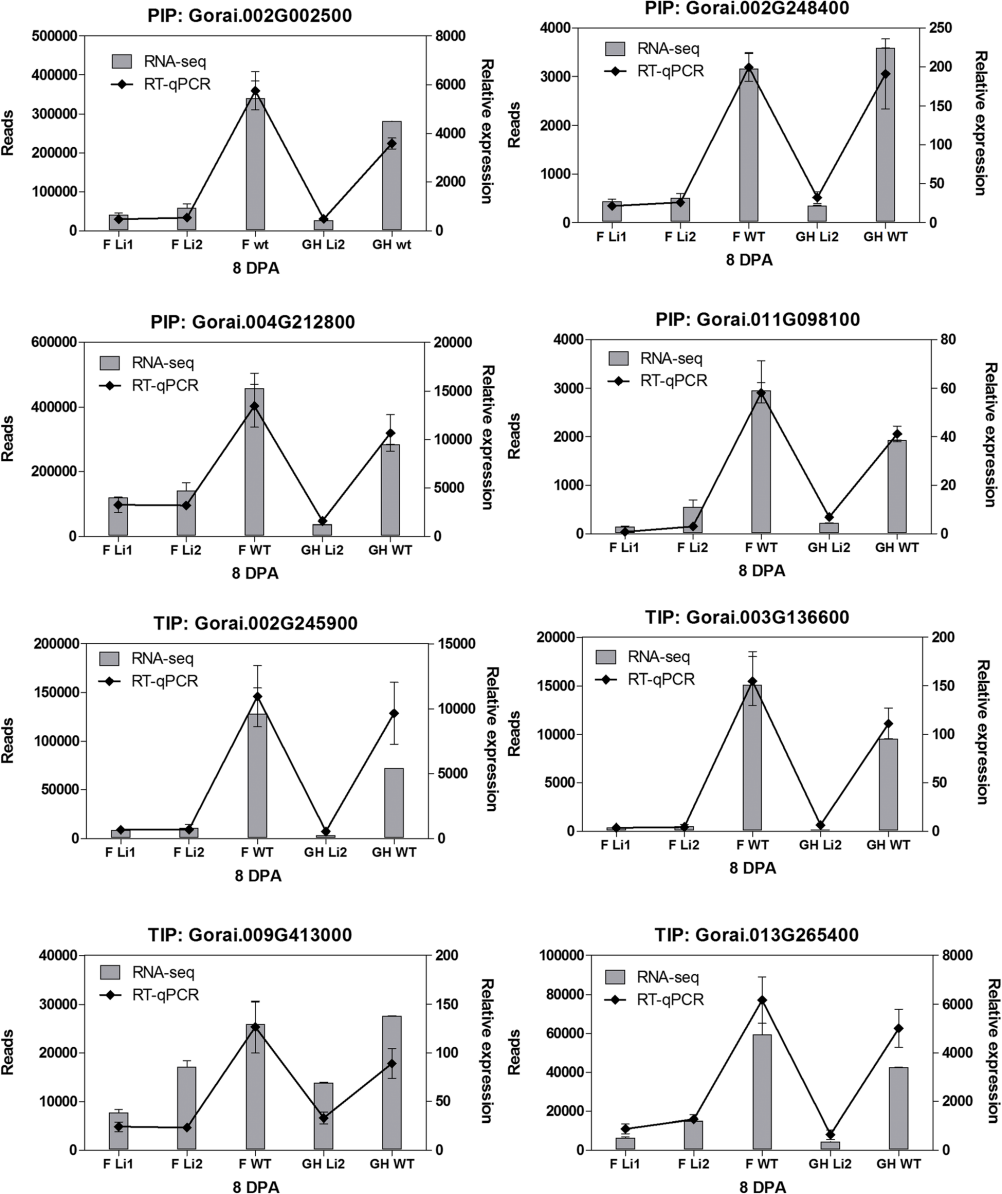
**RNA-seq and RT-qPCR analyses of transcript level of members of the aquaporin family in** ***Li***_***1***_ **,** ***Li***_***2***_ **and WT developing fibers at 8 DPA.** Error bars indicate standard deviation from 2 biological replicates for RNA-seq data and 3 biological replicates for RT-qPCR. Abbreviations: F, field grown plants; GH, greenhouse grown plants; PIP, plasma membrane intrinsic proteins; and TIP, tonoplast intrinsic proteins.

Major intrinsic proteins or aquaporins were one of the most significantly (p < 0.0001) over-represented gene family among down-regulated genes in *Li*_*1*_ – *Li*_*2*_ fibers. Aquaporins facilitate the efficient transport of water and other small molecules across membranes in plants and other organisms [22]. Cotton aquaporins form a large family of proteins phylogenetically divided into five subfamilies including: plasma membrane intrinsic proteins (PIP), tonoplast intrinsic proteins (TIP), NOD26-like intrinsic proteins (NIP), small basic intrinsic proteins (SIP), and the recently identified X (or unrecognized) intrinsic proteins (XIP) [23]. To assess which subfamily members of aquaporins were affected by *Li*_*1*_ – *Li*_*2*_ mutations: first, we conducted phylogenetic analysis of *G. raimondii* genes annotated as aquaporins; and second, evaluated their expression level in *Li*_*1*_ – *Li*_*2*_ developing fibers. The analyzed *G. raimondii* aquaporins clustered into five main clades (marked by empty squares) representing the above mentioned subfamilies (Additional file 3). The members of subfamilies PIP (7 genes), TIP (4 genes) and NIP (2 genes) were down-regulated in *Li*_*1*_ – *Li*_*2*_ developing fibers (marked by black triangle in Additional file 3). The most highly induced aquaporins in WT fibers, for which transcript levels were dramatically reduced in *Li*_*1*_ – *Li*_*2*_ mutants, were tested by RT-qPCR. In most cases results of RT-qPCR analysis were consistent with results of RNA-seq analysis (Figure 3). There were a number of aquaporins which showed increased transcript level only in greenhouse grown *Li*_*2*_ (Additional file 4), indicating interactive response to *Li*_*2*_ mutation and growth conditions. However, relative expression level of those genes was considerably less compared with WT expressed aquaporins as shown in Figure 3 (1,500 reads in greenhouse *Li*_*2*_ induced *vs*. 500,000 reads in WT expressed).

### Osmotic concentrations and solutes in saps of *Li*_*1*_ and *Li*_*2*_ fiber cells

**Figure 4:**
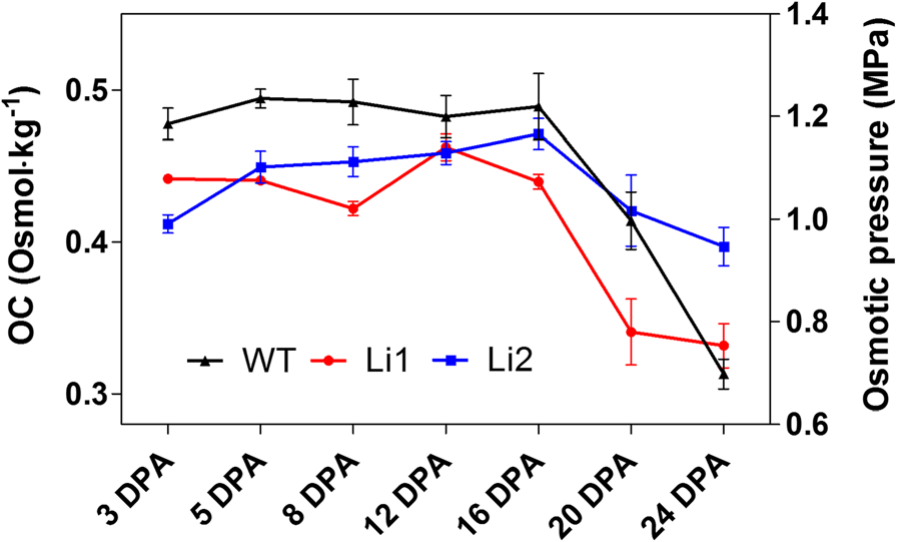
**Osmotic concentration (OC) and the calculated osmotic pressure of the sap of cotton fiber cells.** Cotton fiber cells sap was collected only from field grown plants. Error bars represent standard deviation from 3 biological replicates.

We measured the osmotic concentration and calculated osmotic pressure of the sap of cotton fiber cells. The sap solution represents the average osmotic concentration of the vacuole, the cytoplasm, and the apoplast (i.e. free-space solution) of the fiber cells. In fiber cells the vacuole occupies approximately 90% of the cell volume [4]; therefore the measured osmotic concentration values largely represent the solute concentration of the vacuoles. The calculated osmotic pressure in sap of WT fibers was steadily high during rapid fiber elongation, at 3 – 16 DPA, and significantly dropped during the transition to the cell wall biosynthesis stage (Figure 4). The pattern of osmotic pressure in sap of *Li*_*1*_ fibers was similar with pattern in WT; although the osmotic pressure was significantly lower (p < 0.05) at 3 – 8 DPA. In sap of *Li*_*2*_ fibers the osmotic pressure was significantly lower than in WT at 3 – 5 DPA, but higher at 24 DPA.

**Figure 5:**
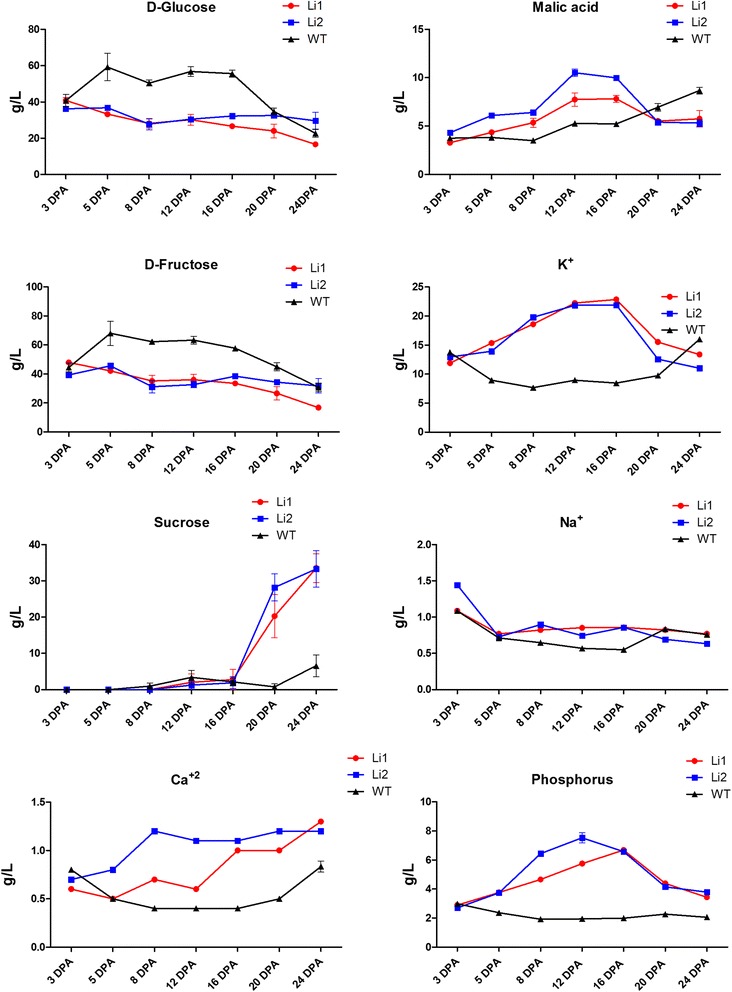
**Concentrations of sugars, malic acid and inorganic ions in saps of developing** ***Li***_***1***_ **,** ***Li***_***2***_ **and WT fibers.** Error bars represent standard deviation: for sugars and malic acid from 3 biological replicates; and for inorganic ions from 3 technical replicates.

Soluble sugars, K^+^, and malate are major active solutes in elongating fibers, to which are often attributed 80% of the fiber sap osmolality [4,24,25]. To assess which osmotic solutes altered in the *Li*_*1*_ and *Li*_*2*_ developing fibers we measured the concentrations of sugars, malic acid, and ions in fiber sap solutions (Figure 5). Concentrations of hexoses (D-glucose and D-fructose) were significantly less in sap of *Li*_*1*_ and *Li*_*2*_ fibers compared to WT during rapid fiber cell expansion (at 5 – 16 DPA). The level of sucrose was low during elongation at 3 – 16 DPA in sap of all near-isogenic lines; however, at 20 – 24 DPA the concentration of sucrose significantly increased in *Li*_*1*_ and *Li*_*2*_, but not in WT fiber. Surprisingly, the concentrations of malic acid and K^+^ were significantly (p < 0.001) higher in sap of *Li*_*1*_ and *Li*_*2*_ fibers comparing to WT during elongation (Figure 5). The concentrations of Na^+^ were not significantly different in saps of *Li*_*1*_, *Li*_*2*_ and WT. We also measured the concentrations of Ca^+2^ and phosphorus, which were significantly higher in saps of mutants compared to WT.

## Discussion

### Experimental design for identification of fiber elongation related genes

In this study we compared the transcriptomes of developing fibers of two short fiber mutants and their WT NIL growing in the field and a greenhouse. The mutated genes of the *Li*_*1*_ and the *Li*_*2*_ are yet to be discovered. A defect in the *Li*_*1*_ gene affected a number of traits (dwarf deformed plants and short fiber phenotype), while the defect in *Li*_*2*_ gene affected only fiber length. Therefore, the *Li*_*1*_ and *Li*_*2*_, most likely, are different types of genes; their alterations interrupt different parts of a complex biosynthetic process, but in both cases cause a short fiber phenotype. Both *Li*_*1*_ and *Li*_*2*_ mutations have an enormous effect on the fiber transcriptomes; the largest source of variability in the fiber transcriptome data was due to mutations (56.4%; Figure 1B). However, altered expression of many genes in *Li*_*1*_ – *Li*_*2*_ transcriptomes can be result of chain-reactions to adverse effects of the causative mutation, and is not necessary directly related to fiber elongation process. Also it is known that many fiber-related genes are environmentally regulated [26]; in our experiment the environmental factor contributed 13.8% to the data variability (Figure 1B). Therefore, to reduce noise in the data we selected common regulated genes between *Li*_*1*_/wt and *Li*_*2*_/wt grown in the field and *Li*_*2*_/wt grown in a greenhouse. This approach allowed the identification of transcripts directly related to fiber elongation process regardless of far downstream effects of the mutations and environmental conditions.

### Gene set enrichment analysis

We found a large number of differentially expressed genes common to both mutants (Figure 2A). To gain insight into biological processes altered by *Li*_*1*_ – *Li*_*2*_ mutations we used MapMan ontology for gene set enrichment analysis. Consistent with our previous microarray study, mitochondrial electron transport functional category was over-represented among up-regulated genes in short fiber mutants [7]. Enrichment of the cell wall functional category was expected among down-regulated genes and described for *Li*_*1*_ and *Li*_*2*_ in our previous reports [5-8]. However, strong down-regulation of major intrinsic proteins in short fiber mutants was not noticed before in our microarray studies, probably due to limitations of microarray techniques. Here, we found that the major intrinsic proteins were the most down-regulated gene family in both short fiber mutants; their role in osmoregulation of *Li*_*1*_ – *Li*_*2*_ fibers is discussed below.

### Osmoregulation in short fiber mutants

The rapid expansion of fiber cells requires high turgor pressure and cell wall relaxation [4,25,27]. The force of turgor pressure is related to the osmotic potential and to the transport coefficient for water uptake [28]. The maintenance of sufficient osmoticum to compensate for dilution effects resulting from the influx of water is an important component of sustainable cell expansion [27]. In the fiber sap of short fiber mutants we detected significantly lower osmotic pressure than in WT. The reduced osmotic pressure in *Li*_*1*_ – *Li*_*2*_ may not be sufficient to maintain rapid and sustainable cell expansion and may cause short fiber phenotype. Soluble sugars, K^+^ and malic acid are considered as major active solutes in rapidly expanding fiber cells [4,24,25]. We detected lower concentrations of glucose and fructose in sap of short fiber mutants than in WT that correlate with lower osmotic pressure, suggesting sugars are the main solutes to positively impact turgor in fiber cells. Sucrose was almost undetectable in mutants and WT fibers during the rapid elongation phase (3 – 16 DPA). In developing fiber cells, sucrose is degraded into hexoses by sucrose synthase in the cytoplasm and acid invertase in the vacuole [24,29,30]. We tested the expression levels of sugars transporters in mutants because their regulation may cause a reduced supply of sugars in developing fibers. However, the number of up-regulated sugars transporters in *Li*_*1*_ – *Li*_*2*_ was higher than down regulated: 4 versus 2 genes, correspondingly (Tables 1 and 2). Therefore, the transport of sugars is unlikely altered in short fiber mutants. In our previous report we observed significant reductions in the levels of detected free sugars, sugar alcohols, sugar acids, and sugar phosphates in the *Li*_*2*_ metabolome; also biological processes associated with carbohydrate biosynthesis were significant down-regulated in the *Li*_*2*_ transcriptome [6]. Consequently, detection of low amount of sugars in sap of *Li*_*1*_ – *Li*_*2*_ fibers might be the result of reduced *de novo* synthesis of sugars in mutants.

**Figure 6:**
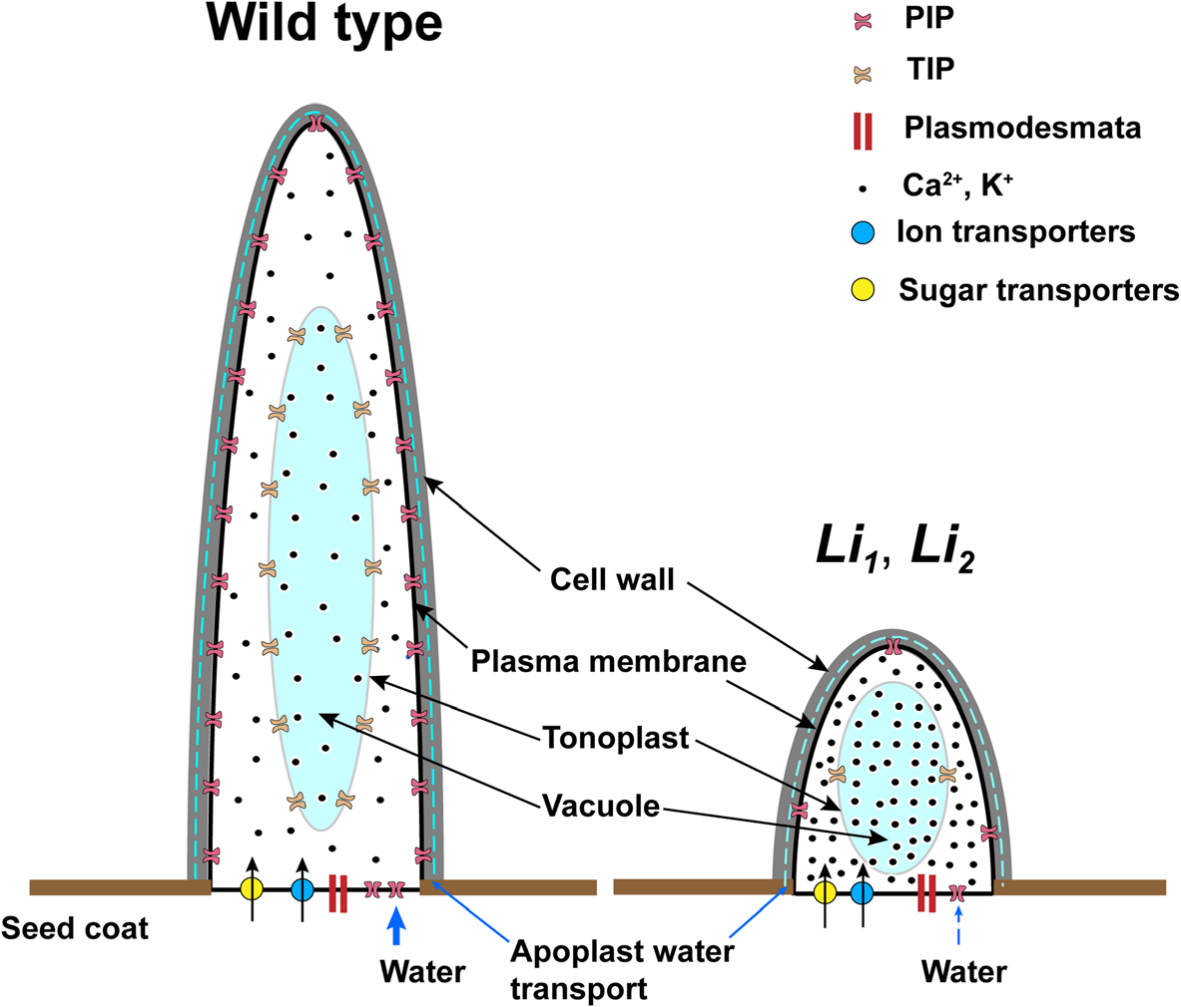
**A possible mechanism of termination of fiber elongation in the** ***Li***_***1***_ **and** ***Li***_***2***_ **mutants.** The high osmotic pressure in fiber cell of WT and high level of expression of aquaporins facilitates influx of water that contributes to the rapid fiber elongation. The higher accumulation of ions in fiber cells of *Li*_*1*_ – *Li*_*2*_ may be the result of limited uptake of water. The reduced influx of water (due to low concentration of sugars and low expression of aquaporins) causes the reduced fiber elongation in the *Li*_*1*_ – *Li*_*2*_ mutants.

The driving force for the transport and accumulation of ions into the protoplast and vacuole is provided by the plasma membrane and vacuolar H^+^-ATPases [27,31]. We did not detect the plasma membrane and vacuolar H^+^-ATPases among common *Li*_*1*_ – *Li*_*2*_ up-regulated or down-regulated pools of genes. Numbers of calcium, potassium and other metal transporters were not significantly different between pools of up-regulated and down-regulated genes in short fiber mutants; except for sulphate and phosphate transporters which were present among down-regulated genes only (Tables 1 and 2). Thus, ion transport in *Li*_*1*_ – *Li*_*2*_ is unlikely to be affected by the mutations and proceeds normally as in wild type plants. The higher concentrations of malic acid, K^+^ and other inorganic ions detected in sap of *Li*_*1*_ – *Li*_*2*_ can be explained by reduced influx of water into fiber cells of mutants (Figure 5). Since malic acid and K^+^ (major osmotic solutes) cannot restore the balance of water uptake into developing *Li*_*1*_ – *Li*_*2*_ fibers, there is another factor, which might be crucial for osmoregulation of cotton fibers – the major intrinsic proteins (Figure 6).

The major intrinsic proteins or aquaporins were the most overrepresented gene family among down-regulated genes in both short fiber mutants (Table 2). The expression level of some members of PIPs and TIPs at 8 DPA of fiber development in WT was enormous, up to 500,000 reads (Figure 3). It has been indicated in a number of studies that the osmotic water permeability (or hydraulic conductivity) is controlled by the activity of aquaporins. For instance, Javot and co-authors showed that Arabidopsis PIP2;2 is highly expressed in several root cell types, and that, by comparison to WT plants, the hydraulic conductivity of corresponding knock-out mutants (*pip2;2*) was reduced by 14% [32]. The hydraulic conductivity of *pip1;2* mutants and *pip2;1* and *pip2;2* double mutants was decreased by 20% and 40% respectively, compared to that of WT [33,34]. A link between aquaporins and cell growth has also been shown in different species. Virus-induced silencing of rose *PIP2;1* resulted in a reduction in size of cells and petal expansion [35]. Over-expression of a cauliflower TIP1-GFP fusion in tobacco suspension cells or of ginseng TIP in Arabidopsis leaves led to an increase in cell size [36,37]. Vacuole regeneration and cell expansion were accelerated in protoplast prepared from BY-2 cells over-expressing the NtTIP1;1 [38]. Knockdown of expression of *GhPIP2* genes by RNA interference in *G. hirsutum* markedly inhibited fiber elongation [39]. Thus, the reduced expression of aquaporins in short fiber mutants may reduce the influx of water into fiber cells and slow down the elongation process (Figure 6).

## Conclusions

Here, we used an RNA-seq approach to determine common fiber elongation related genes in developing fibers of *Li*_*1*_ and *Li*_*2*_ mutants growing in the field and a greenhouse. We found that the aquaporins were the most down-regulated gene family in both short fiber mutants. The osmolality and concentrations of soluble sugars were less in saps of *Li*_*1*_ – *Li*_*2*_, whereas the concentrations of malic acid, K^+^ and other detected ions were significantly higher in saps of mutants than in WT. These results suggest that higher accumulation of ions in fiber cells, reduced osmotic pressure and low expression of aquaporins, may contribute to the cessation of fiber elongation in *Li*_*1*_ and *Li*_*2*_ short-fiber mutants.

## Methods

### Plant materials

Two mutant lines *Li*_*1*_ and *Li*_*2*_ in a near-isogenic state with the WT upland cotton line DP5690 were developed in a backcross program at Stoneville, MS as described before [5,8]. The growing period for the greenhouse grown *Li*_*2*_ plants was between October, 2009 and March, 2010; planting and growth conditions were previously described [5]. For the field grown plants, a total of 150 *Li*_*1*_, 100 *Li*_*2*_, and 100 WT plants were grown in a field at the USDA-ARS Southern Regional Research Center, New Orleans, LA in the summer of 2013. All samples of the same developmental stage were tagged and collected on the same day. Cotton bolls were harvested at 3, 5, 8, 12, 16, 20, and 24 DPA. Bolls were randomly separated into 3 replicates with 15–30 bolls per replicate.

### RNA isolation and reverse transcription quantitative polymerase chain reaction (RT-qPCR)

Total RNA was isolated from detached fibers [40] using the Sigma Spectrum Plant Total RNA Kit (Sigma-Aldrich, St. Louis, MO) with the optional on column DNase1 digestion according to the manufacturer’s protocol. The concentration of each RNA sample was determined using a NanoDrop 2000 spectrophotometer (NanoDrop Technologies Inc., Wilmington, DE). The RNA quality for each sample was determined by RNA integrity number (RIN) using an Agilent Bioanalyzer 2100 and the RNA 6000 Nano Kit Chip (Agilent Technologies Inc., Santa Clara, CA) with 250 ng of total RNA per sample. RNA from each of the above mentioned time-points was used for RT-qPCR analysis. A detailed description of reverse transcription, qPCR and expression analysis was previously reported [9]. Sequences of primers used for qPCR are listed in Additional file 5.

### Deep sequencing and differential gene expression

Library preparation and sequencing were performed by Data2Bio LLC (Roy J. Carver Co-Laboratory, Ames, Iowa). The libraries were sequenced using 101 cycles of chemistry and imaging, resulting in paired end (PE) sequencing reads with length of 2 × 101 bp. For the greenhouse grown *Li*_*2*_ plants samples were sequenced in two biological replicates (sequencing and data were described elsewhere [9]). For the field grown *Li*_*1*_, *Li*_*2*_ and WT plants RNA samples from cotton fiber at 8 DPA were sequenced in three biological replicates. The 8 DPA was chosen because it is the peak of fiber elongation phase according to our earlier studies [5,6,8]. RNA-seq expression analysis was conducted following the PolyCat pipeline [20]. Briefly, all reads were aligned to the JGI *Gossypium raimondii* reference genome [18], then the PolyCat software assigned each categorizable read to either the A or D subgenome based on an index of homeoSNPs. We followed two adjustments previously described [10], particularly: 1) we only counted exonic reads; 2) we used the ratio of A-assigned to D-assigned reads to proportionally divide the total number of mapped reads for each gene which ensures that unassigned reads contribute to the total expression of genes. The data normalization and ANOVA process were conducted as previously described [9]. Principal component analysis (PCA) was conducted using JMP Genomics 7 software (SAS Institute Inc., Cary, NC, USA). Transcript data for each sample were used as continuous variables. Principal variance component analysis (PVCA) reduces the dimensionality of the data set with PCA, and then fits a mixed linear model to each principal component to partition variability with variance components analysis (VCA). A summary of variance components across all principal components is constructed as a weighted average of the individual estimates, using eigenvalues as weights [41].

### Osmotic concentration measurement of cotton fiber saps

Saps were collected from cotton fiber cells from the each time point mentioned above. From short fiber ovules fibers were collected by shaking ovules frozen in liquid nitrogen. Fibers from long fiber ovules were pulled off by forceps. For each replicate, about 200 mg of frozen fibers were thawed on ice. Sap was separated by centrifugation at 5,000 g for 1 min [30]. The osmotic concentration of cotton fiber sap was measured using a vapour pressure osmometer, VAPRO-5600 (WESCOR INC., South Logan, UT, USA) in three biological replicates as previously described [30]. The osmotic pressure (MPa) was calculated from the osmotic concentration using the equivalence 2.44 MPa per 1 Osmol∙kg^−1^ (25°C) [42].

### Measurement of sugars and malic acid concentrations in cotton fiber saps

Concentrations of sucrose, D-fructose and D-glucose in cotton fiber saps were measured using enzyme assay kit K-SUFRG (Megazyme International, Ireland) according to the manufacturer’s instructions. Sap samples were diluted 50 times in water for sugar determination. Concentration of L-malic acid in cotton fiber sap was measured using enzyme assay kit K-LMALR (Megazyme International, Ireland) according to the manufacturer’s instructions. Sap samples were diluted 5 times in water for malic acid determination.

### Measurement of ions in cotton fiber saps

Potassium, sodium, calcium and phosphorous contents were determined using Prodigy High Dispersion Inductively Coupled Plasma Optical Emission Spectrometer (ICP-OES, Teledyne Leeman Labs). A series of KNO_3_, Na_2_CO_3_, CaO and H_3_PO_4_ concentrations (diluted in 2% nitric acid) were used as standards. The nitric acid digestion of sap samples was conducted according to method reported elsewhere [43]. Particularly, 1 ml of pure grade 20% nitric acid was added to 200 μl of sap in an acid-washed plastic test tube. The sealed tube containing sap acid solution was incubated at 65°C for at least 12 hours. The digested sap solution was diluted with 2% nitric acid and analyzed with ICP-OES in three technical replicates.

### Availability of supporting data

RNA-seq data from developing fibers (at 8 DPA) of field grown plants from two short-fiber mutants, *Li*_*1*_ and *Li*_*2*_ and their NIL *G. hirsutum* DP5690 are available in the NCBI SRA archive (accession # PRJNA273732).

## Additional files

Additional file 1:
**The results of mapping reads.**
Additional file 2:
**The ANOVA analysis of RNA-seq data.**
Additional file 3:
**Phylogenetic analysis of** ***G. raimondii*** **aquaporins.** The evolutionary analysis of *G. raimondii* aquaporins was conducted in MEGA6 [44] using the Neighbor-Joining method [45]. The percentage of replicate trees in which the aquaporin genes clustered together in the bootstrap test (1000 replicates) are shown next to the branches [46]. The tree is drawn to scale, with branch lengths in the same units as those of the evolutionary distances used to infer the phylogenetic tree. The evolutionary distances were computed using the Poisson correction method [47] and are in the units of the number of amino acid substitutions per site. The analysis involved 59 amino acid sequences. All positions containing gaps and missing data were eliminated. There were a total of 48 positions in the final dataset. Sequence for the aquaporin GhPIP2;6 previously characterized *G. hirsutum* can be found in GenBank database under the following accession number: FJ646597. Additional file 4:
**RNA-seq and RT-qPCR analyses of transcript level of members of the aquaporin family which were induced in** ***Li***_***2***_ **in greenhouse growth environment.** Error bars indicate standard deviation from 2 biological replicates for RNA-seq data and 3 biological replicates for RT-qPCR. Abbreviations: F, field grown plants; GH, greenhouse grown plants; PIP, plasma membrane intrinsic proteins; and PIP, tonoplast intrinsic proteins. Additional file 5:
**Sequences of primers used for RT-qPCR expression analysis.**

## Abbreviations

*Li*_*1*_: Ligon lintless-1
*Li*_*2*_: Ligon lintless-2
DPA: Day post-anthesis
NILs: Near-isogenic lines
WT: Wild type
PIP: Plasma membrane intrinsic protein
TIP: Tonoplast intrinsic protein
