# Supplementary figures and images for: RNA-seq analysis of short fiber mutants Ligon-lintless-1 (Li1) and – 2 (Li2) revealed important role of aquaporins in cotton (Gossypium hirsutum L.) fiber elongation
Source: BMC Plant Biol. 2015 Feb 27;15:65. doi: 10.1186/s12870-015-0454-0 (PMC4352256; doi:10.1186/s12870-015-0454-0)

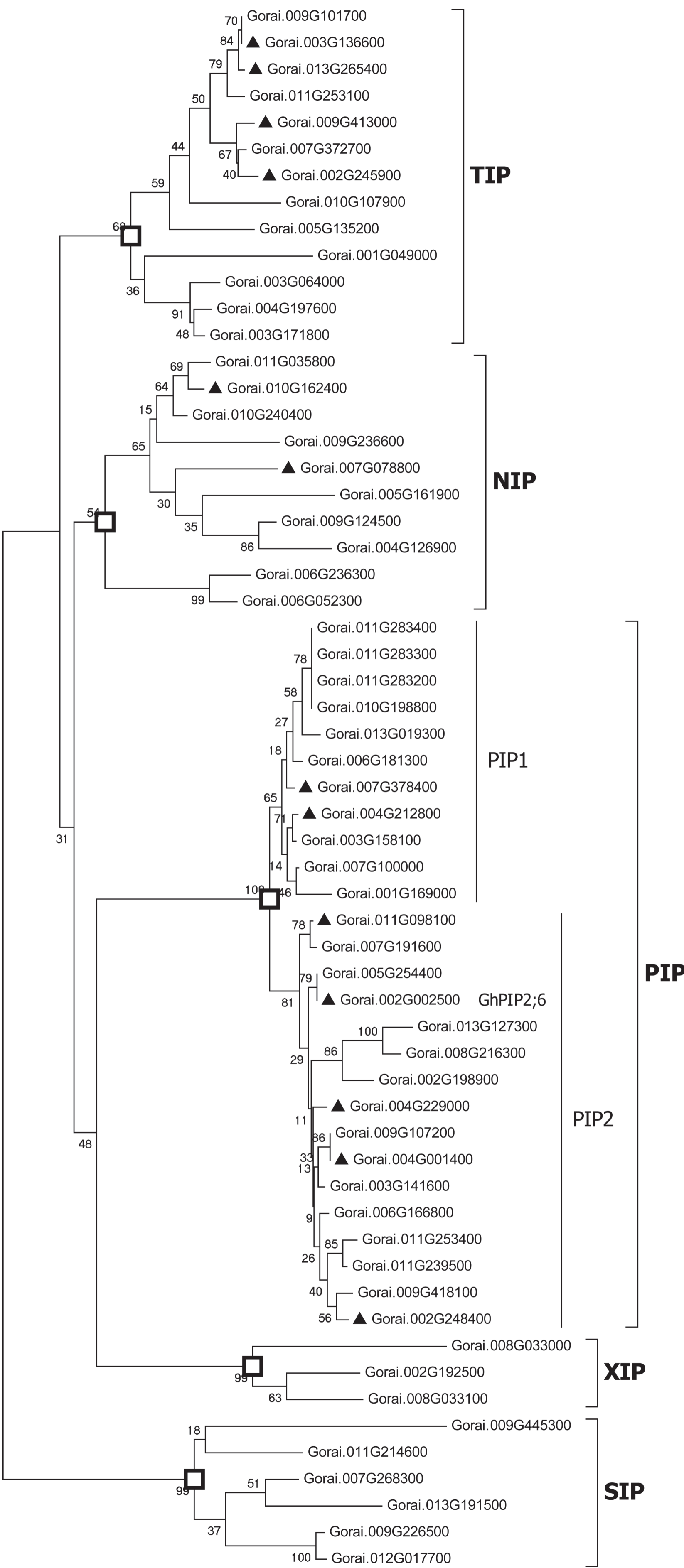

Supplement: Additional file 3: — Phylogenetic analysis of G. raimondii aquaporins. The evolutionary analysis of G. raimondii aquaporins was conducted in MEGA6 [44] using the Neighbor-Joining method [45]. The percentage of replicate trees in which the aquaporin genes clustered together in the bootstrap test (1000 replicates) are shown next to the branches [46]. The tree is drawn to scale, with branch lengths in the same units as those of the evolutionary distances used to infer the phylogenetic tree. The evolutionary distances were computed using the Poisson correction method [47] and are in the units of the number of amino acid substitutions per site. The analysis involved 59 amino acid sequences. All positions containing gaps and missing data were eliminated. There were a total of 48 positions in the final dataset. Sequence for the aquaporin GhPIP2;6 previously characterized G. hirsutum can be found in GenBank database under the following accession number: FJ646597. [file 12870_2015_454_MOESM3_ESM.pdf]

PIP: Gorai.005G254400

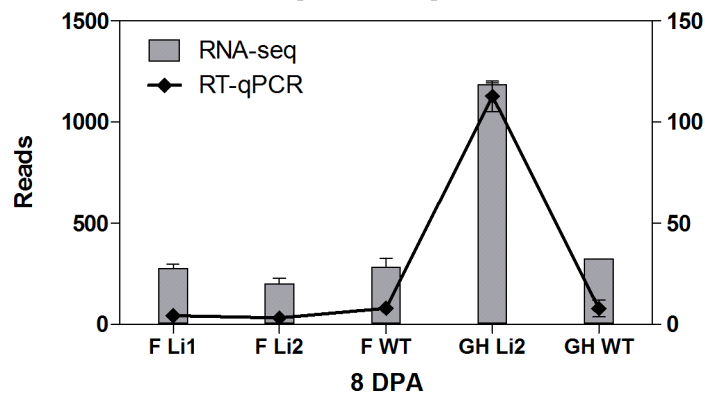

PIP: Gorai.007G100000

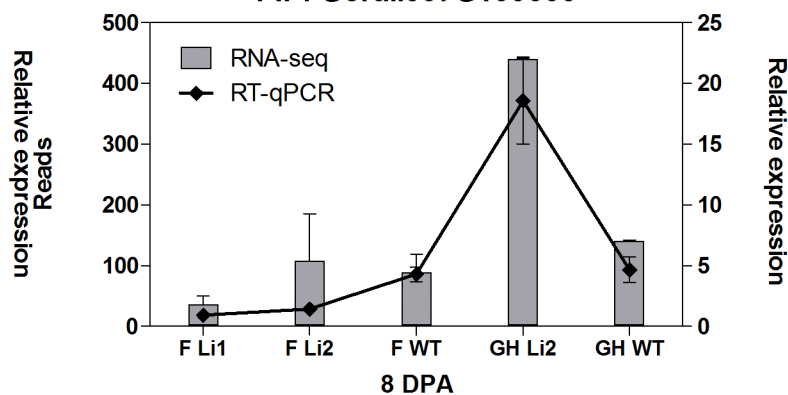

PIP: Gorai.008G033100

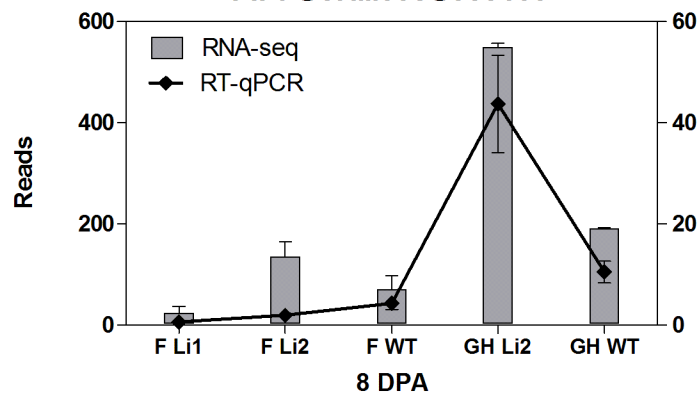

PIP: Gorai.009G418100

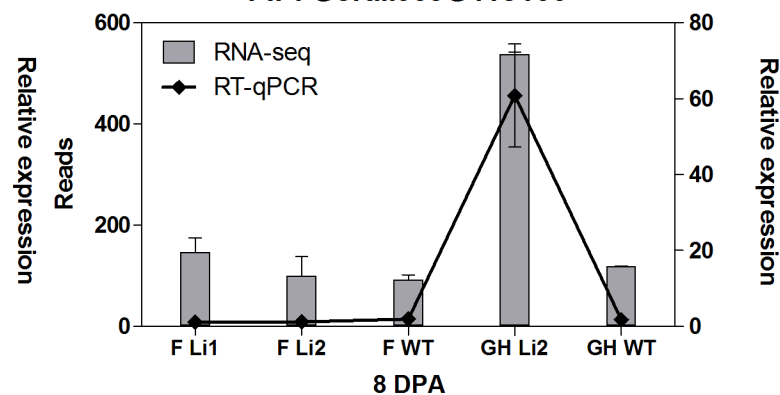

PIP: Gorai.010G198800

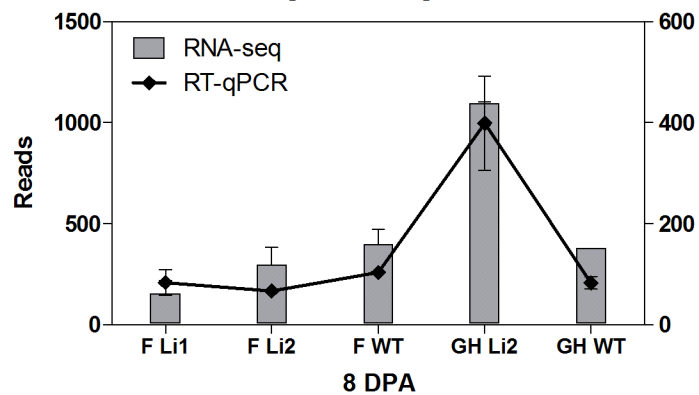

PIP: Gorai.011G239500

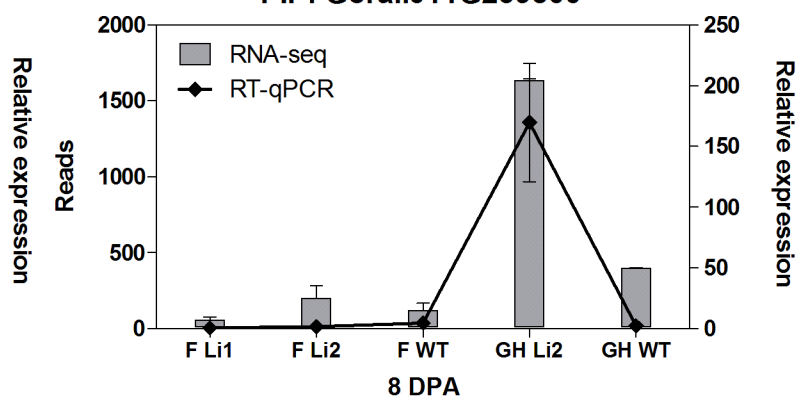

PIP: Gorai.006G166800

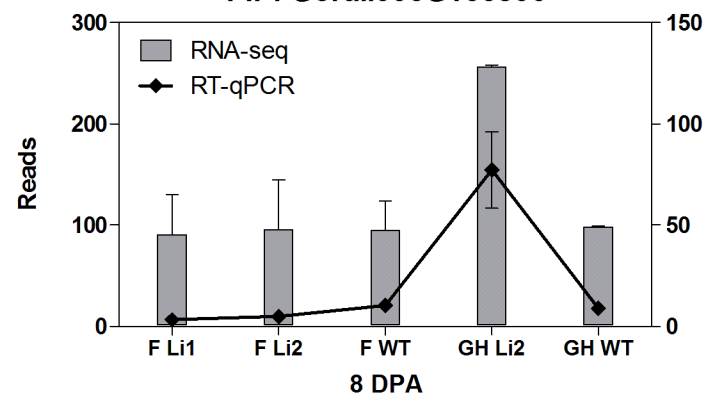

PIP: Gorai.011G253400

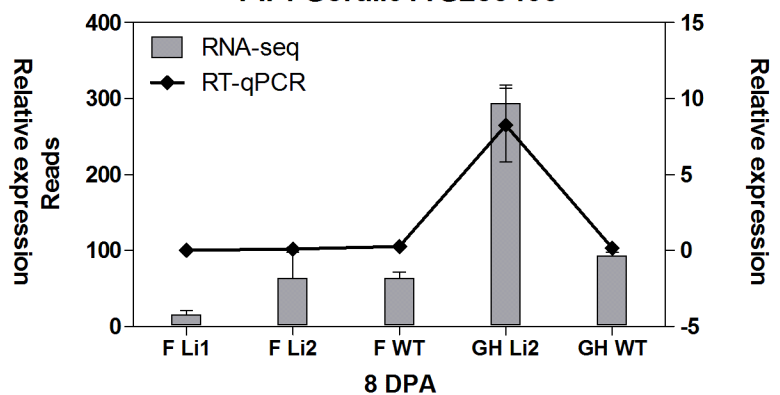

Supplement: Additional file 4: — RNA-seq and RT-qPCR analyses of transcript level of members of the aquaporin family which were induced in Li 2 in greenhouse growth environment. Error bars indicate standard deviation from 2 biological replicates for RNA-seq data and 3 biological replicates for RT-qPCR. Abbreviations: F, field grown plants; GH, greenhouse grown plants; PIP, plasma membrane intrinsic proteins; and PIP, tonoplast intrinsic proteins. [file 12870_2015_454_MOESM4_ESM.pdf]
